# The prevalence of insomnia disorder in inpatient psychiatric care

**DOI:** 10.1007/s00406-026-02285-z

**Published:** 2026-06-26

**Authors:** Elisabeth Hertenstein, Heidi Danker-Hopfe, Christian Mikutta, Eva Allenbach, Peggy Schmidt, Marian Spath, Elmar Großkurth, Carlotta L. Schneider, Lukas Frase, Claudia Schilling, Michael Schredl, Elisabeth Anzenberger, Robert Göder, Silja Weber, Christine Norra, Christian Sander, Nicole Mauche, Christian Makiol, Maria Strauß, Thomas C. Wetter, Franziska C. Weber, Christoph Nissen

**Affiliations:** 1 Department of Psychiatry, Faculty of Medicine, University of Geneva, Rue de Lausanne 20, 1201 Geneva, Switzerland; 2 University Hospital of Psychiatry and Psychotherapy, University of Bern, Bern, Switzerland; 3 Department of Psychiatry and Neurosciences, Competence Centre of Sleep Medicine, Charité – Universitätsmedizin Berlin, Freie Universität Berlin and Humboldt Universität zu Berlin, Campus Benjamin Franklin, Berlin, Germany; 4 Privatklinik Meiringen, Meiringen, Switzerland; 5 Department of Psychosomatic Medicine and Psychotherapy, Medical Center, Faculty of Medicine, University of Freiburg, Freiburg, Germany; 6 Department of Psychiatry and Psychotherapy, Medical Center, University of Freiburg-Faculty of Medicine, Freiburg, Germany; 7 Central Institute of Mental Health, Medical Faculty Mannheim/Heidelberg University, Mannheim, Germany; 8 Department of Psychiatry and Psychotherapy, University Hospital Schleswig-Holstein, Campus Kiel, Kiel, Germany; 9 Psychiatry Psychotherapy Psychosomatics, Medical Faculty Bochum, LWL Hospital Paderborn, Paderborn, Germany; 10 Department of Psychiatry and Psychotherapy, University of Leipzig Medical Center, Leipzig, Germany; 11 Department of Psychiatry and Psychotherapy, Faculty of Medicine, Leipzig University, Leipzig, Germany; 12 Department of Psychiatry and Psychotherapy, University of Regensburg, Regensburg, Germany; 13 Department of Psychiatry, Geneva University Hospitals (HUG), Geneva, Switzerland

## Abstract

Sleep and mental health are intricately linked. Insomnia – defined as a dissatisfaction with sleep quantity or quality – is highly prevalent among patients with psychiatric disorders, exerting a negative impact on treatment outcome. However, the prevalence of insomnia on a symptom and diagnosis level (insomnia disorder) has not systematically been assessed in inpatient psychiatric care. The aim of this study was to systematically investigate the prevalence of insomnia symptoms and insomnia disorder according to Diagnostic and Statistical Manual of Mental Disorders, Fifth Edition (DSM-5) criteria in inpatient psychiatric care. Comprehensive diagnostic interviews were conducted by trained clinicians in four German and two Swiss psychiatric hospitals. A total of 427 inpatients with psychiatric disorders across diagnostic groups were included (225 female, 200 male, 2 non-binary, mean age 40.8 ± 15.1 years, mean duration of psychiatric illness 8.6 ± 10.6 years). The prevalence of comorbid insomnia disorder, according to DSM-5 criteria, was 30.4% (95% CI [26.0%; 34.8%]). Additionally, 38.4% of patients (95% CI [33.8%; 43.0%]) reported regular insomnia symptoms but did not fulfill disorder criteria. Together, 68.8% of the sample (95% CI [66.4%; 73.2%]) were regularly affected by insomnia complaints. The prevalence of insomnia disorder was independent of age and diagnostic group, but significantly higher in women than in men (Fisher’s exact test: *p* = 0.006, OR = 1.8, 95% CI [1.2; 2.8]). The current study is, to our knowledge, the first interview-based systematic investigation of the prevalence of insomnia disorder in inpatient psychiatric care. The high prevalence of around one third of patients is a call for action to implement a systematic diagnostic evaluation and state-of-the-art treatment of insomnia in routine psychiatric care. Of note, recent work demonstrates that adaptations of cognitive behavioral therapy for insomnia (CBT-I) – the first-line treatment according to guidelines—are feasible in inpatient psychiatric care. These interventions might have the potential to reduce the overprescription of hypnotic medication and to improve sleep and health.

## Introduction

Mental disorders are highly prevalent and represent a significant global burden of disease [1]. They are closely intertwined with sleep, and many patients with mental disorders report a dissatisfaction with sleep quantity or quality (insomnia).

According to current diagnostic systems, the Diagnostic and Statistical Manual of Mental Disorders, Fifth Edition (DSM-5) and the International Classification of Diseases, 11th Revision (ICD-11) [2, 3], *insomnia disorder* is defined as a complaint of reduced sleep quantity or quality, associated with daytime impairment, occurring on several nights per week for at least three months. For an insomnia disorder diagnosis, the complaint must persist despite adequate opportunities for sleep and appear to not be fully explained by another sleep, psychiatric or physical disorder, or by substance use.

Given the complex interaction of insomnia and mental health problems, a clinically meaningful and reasonably robust definition of insomnia disorder (diagnosis level) emerges when insomnia symptoms occur, at least in part, independently of another disorder (i.e., they precede or persist beyond another disorder episode), or when the severity of the sleep complaint appears not to be sufficiently explained by the co-occurring disorder. Beyond the diagnostic level, insomnia complaints often co-occur with a wide range of psychiatric disorders and can be described as *insomnia symptoms* (without fulfilling diagnostic criteria at the disorder level).

The shift from conceptualizing insomnia solely as a symptom according to traditional psychiatric views, to recognizing insomnia as a distinct disorder in DSM-5 and ICD-11, is supported by the following arguments. Insomnia can occur independently in the absence of mental disorders yet increases the risk of developing psychiatric disorders [4]. Moreover, insomnia often persists even after remission of an episode of a psychiatric disorder and increases the risk for relapse [5]. Importantly, treating insomnia can reduce the risk of developing a mental disorder, improve treatment outcome during episodes of psychiatric disorders (beyond sleep), and lower the risk for relapse after remission [6]. While the underlying mechanisms remain largely unclear, these observations indicate sufficient independency and treatment potential to fulfill disorder level criteria (comorbidity principle). Together, systematically diagnosing and treating insomnia disorder has the potential to improve sleep and mental health. Of note, according to current treatment guidelines [7–9], cognitive behavioral therapy for insomnia (CBT-I) represents the first line treatment for adults, including those with psychiatric disorders, but is insufficiently implemented into routine clinical care. Still, overmedication is frequent, associated with the risk of side effects and the development of tolerance and dependency [10, 11].

Characterizing the prevalence of insomnia symptoms and insomnia disorder is of high importance for diagnostic processes, treatment optimization and the allocation of resources in the health care system. In outpatients with psychiatric disorders, prevalence estimates based on interviews have been published indicating a high prevalence of both insomnia symptoms and insomnia disorder. On average, one third of treatment-seeking outpatients with affective disorders, anxiety disorders or schizophrenia spectrum disorders fulfilled diagnostic criteria for comorbid insomnia disorder, and the prevalence of insomnia symptoms was even higher especially in those with depression [12]. A recently published meta-analysis on the prevalence of insomnia disorder in the general population underlines the importance of using structured diagnostic interviews by trained raters [13]. While the authors reported a pooled prevalence rate of 12.4% in studies using diagnostic interviews (confidence interval: 9% to 16.8%), studies estimating the prevalence from different self-rating questionnaires had highly variable results, with prevalence rates between 7.5% and 32.3%, which is not sufficiently robust for decision making in the healthcare system. Therefore, the authors of the meta-analysis recommend using standardized, structured in-person interviews for diagnosis and prevalence estimates.

To date, the prevalence of insomnia symptoms and insomnia disorder in inpatients with psychiatric disorders has not systematically been investigated. This investigation is important since inpatients differ from outpatients, with typically higher symptom severity, more acute clinical problems and a different distribution of diagnostic groups. Particularly, an inpatient stay represents a window of opportunity for defining diagnosis and treatment pathways.

The aim of the current study was to investigate the prevalence of insomnia symptoms and insomnia disorder, based on standardized structured clinical interviews by trained clinicians, in inpatient psychiatric care.

## Methods

### Overview

The study has been initiated by the Sleep section of the German Association for Psychiatry, Psychotherapy and Psychosomatics (DGPPN) and conducted in six psychiatric hospitals in Switzerland and Germany (University Psychiatric Services Bern and the Psychiatric Hospital Meiringen in Switzerland, as well as the Departments of Psychiatry at the University Medical Centers in Freiburg, Kiel, Leipzig and Mannheim in Germany). Data analysis was performed centrally at the Charité Berlin.

This study was approved by the local ethics committees at the participating study sites. The study protocol had been preregistered prior to the start of the study (www.clinicaltrials.gov, NCT05408013). The study was funded by intramural funds of the study sites.

### Recruitment procedure

Data was collected between January 2021 and March 2025. The recruitment of study participants was consecutive. At each study site, all newly admitted patients were registered on a list each day. This list was available for the study clinicians. From this list, patients who were clearly not eligible (involuntary stay, under custody) were removed by the clinicians. Per week, 1–15 patients (depending on the clinicians’ capacity) were randomly selected from the patients remaining on the list. Randomization was performed by participating clinicians at the respective study sites using a random number table, before obtaining informed consent. There was no stratification applied. Clinicians were blinded, that is they could not foresee or influence which patients would be selected.

The selected patients were asked to participate in the study and, if they gave written informed consent, screened for the inclusion and exclusion criteria.

**Fig. 1:**
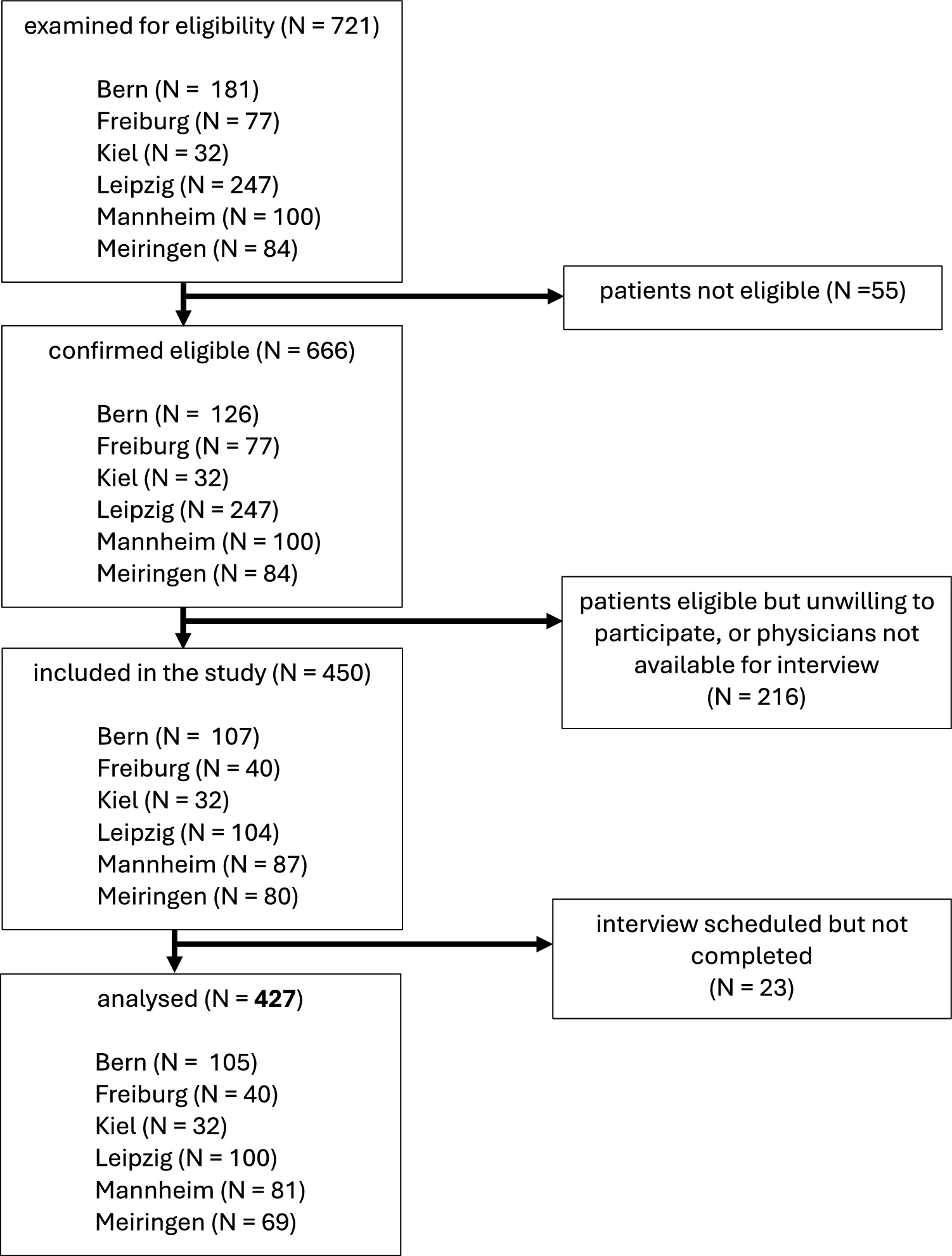
Participant flow

The inclusion criteria were the following: Diagnosis of a psychiatric disorder according to ICD-10 (across all diagnostic entities), currently in inpatient treatment in one of the participating study centers, and age of 18 years or above. Exclusion criteria were an involuntary stay at the clinic, patients under custody and an inability to provide written informed consent/participate in the study procedures. The numbers of screened and included patients is shown in the flowchart (Fig. 1).

Figure 1 depicts the numbers of patients examined for eligibility, confirmed eligible, included in the study (informed consent), and analysed (interview completers).

Included patients were interviewed in person by a clinician (assistant or senior board-certified physician in psychiatry, or licensed psychotherapist). All participating clinicians were specifically trained to perform the comprehensive structured diagnostic interview for insomnia symptoms and disorder (duration 30–45 min). Online meetings between sites were conducted to ensure comparable procedures and evaluation. The interview covered the diagnostic criteria for insomnia disorder according to DSM-5 as depicted in Table 1. Question “H” about a co-existing psychiatric or somatic disorder explaining the insomnia is particularly critical in the context of this study. To clarify this point and to allow for a consistent, standardized approach across the six study centers, three additional questions beyond the diagnostic criteria were added to guide interviewers in their decisions. These questions were about temporal dependence or independence of insomnia and the main psychiatric disorder leading to inpatient admission, the severity of insomnia being explained or not explained by the severity of the other disorder, and a cognitive and emotional focus of the patient on insomnia symptoms. Patients who fulfilled diagnostic criteria for insomnia disorder according to DSM-5, including criterion “H”, were given a diagnosis of insomnia disorder. Patients who responded “yes” to criterion “A” (complaint of dissatisfaction with sleep quantity or quality), but did not fulfill all criteria for insomnia disorder, were allocated to the “insomnia symptoms” group.

**Table 1:** Diagnostic criteria for insomnia disorder according to DSM-5

A	Predominant complaint of dissatisfaction with sleep quantity or quality, associated with one (or more) of the following symptoms: difficulty initiating sleep, difficulty maintaining sleep, or early morning awakening
B	The sleep disturbance causes clinically significant distress or impairment in social, occupational, educational, academic, behavioral, or other important areas of functioning
C	The sleep difficulty occurs at least 3 nights per week
D	The sleep difficulty is present for at least 3 months
E	The sleep difficulty occurs despite adequate opportunity for sleep
F	The insomnia is not better explained by and does not occur exclusively during the course of another sleep–wake disorder
G	The insomnia is not attributable to the physiological effects of a substance
H	Coexisting mental disorders and medical conditions do not adequately explain the predominant complaint of insomnia

In addition to the diagnostic criteria, the interview included questions about clinical and demographic characteristics, past medical and psychotherapeutic treatment of insomnia, current pharmacological treatment, known sleep disorders other than insomnia as well as substance use. These questions were answered through the patient interview in combination with a review of patient records. For patients who had more than one psychiatric diagnosis, the main psychiatric diagnosis used for further analysis was defined as the diagnosis mainly responsible for the current inpatient stay.

To further characterize the sample, the patients also filled-out several self-rating questionnaires, namely the Insomnia Severity Index (ISI) assessing the severity of insomnia including nighttime and daytime symptoms [14], the Pittsburgh Sleep Quality Index (PSQI) [15], the Epworth Sleepiness Scale (ESS) [16] assessing subjective daytime sleepiness, the Patient Health Questionnaire (PHQ-9) [17] assessing the severity of depressive symptoms, and the Fatigue Severity Scale (FSS) [18].

### Statistics

The primary outcome was the prevalence of insomnia disorder, measured per patient at one time point during an interview with a clinician, as preregistered on www.clinicaltrials.gov (NCT05408013). Additionally, the prevalence of insomnia symptoms not fulfilling diagnostic criteria for insomnia disorder was investigated. Insomnia symptoms were here defined as the presence of a predominant complaint of a dissatisfaction with sleep quality or quantity (“yes” to criterion A, Table 1).

An a priori sample size consideration revealed that with a sample size of n = 500 patients prevalences ranging from 15 to 25% can be estimated with a 95% confidence interval ranging from [11.9%; 18.1%] to [21.2%; 28.8%].

Exploratory analyses were performed to investigate differences in prevalence between subgroups related to diagnostic group, age and gender. For statistical comparisons between diagnostic groups, patients were divided into six groups: F1 (substance use disorders), F2 (psychotic disorders), F3 (mood disorders), F4 (neurotic and psychosomatic disorders), F6 (personality disorders), and others including organic psychiatric disorders, eating disorders, and developmental disorders. The “others” category was used due to the low number of patients in these diagnostic categories. Age was investigated using a median split resulting in a younger age group (age below median) and an older age group (age above median). For statistical comparisons between gender groups, non-binary patients were omitted due to their low number (n = 2). To investigate potential differences in the prevalence between different diagnostic groups, age (median split) and gender (male / female), Loglikelihood Ratio Chi square test and Fisher’s exact test were applied. All tests were conducted with a two-sided error probability of 0.05. Odds Ratios were calculated to quantify the strength of potential distribution differences. All statistical analyses were performed using SAS 9.4.

## Results

### Prevalence of insomnia disorder diagnosis in the total sample and subgroups

**Fig. 2:**
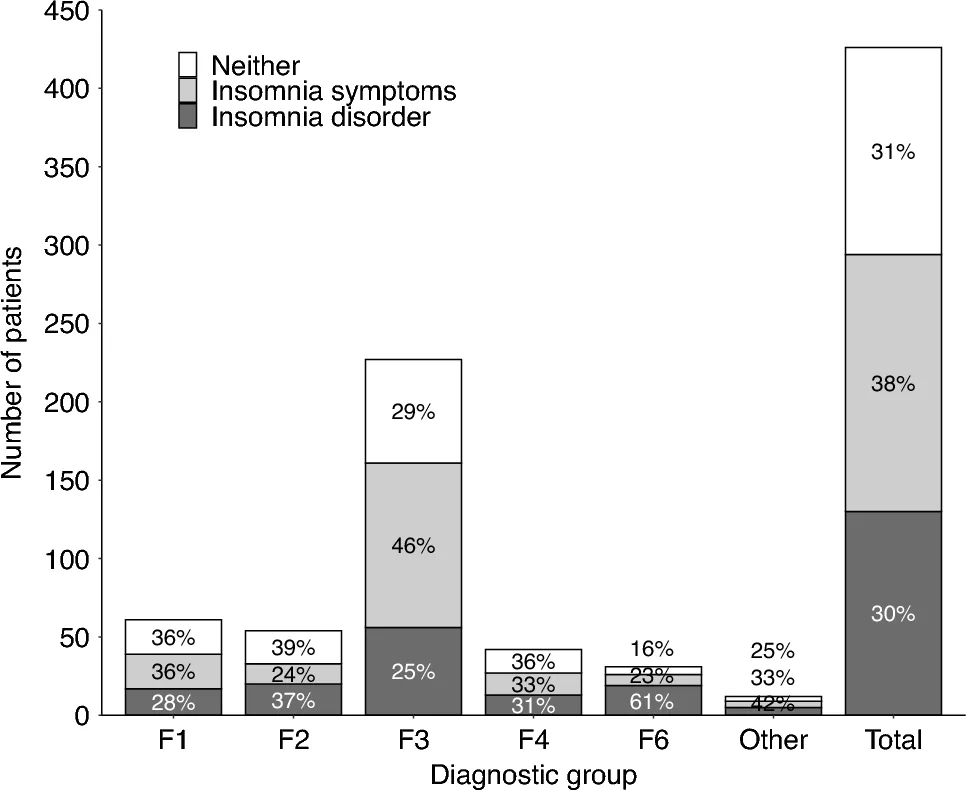
Prevalence of insomnia disorder diagnosis and insomnia symptoms per diagnostic category and in the total sample. F1 substance use disorders, F2 psychotic disorders, F3 mood disorders, F4 neurotic and psychosomatic disorders, F6 personality disorders, and others (including organic psychiatric disorders, eating disorders, and developmental disorders)

The prevalences of insomnia disorder and insomnia symptoms are shown in Fig. 2 for the total sample and per diagnostic group. The prevalence between diagnostic groups was statistically significantly different (likelihood-ratio chi square test: p = 0.0041).

As the main finding of the current study, the prevalence of insomnia disorder observed in the total sample (across diagnostic groups) was 30.4% (130 out of 427 patients, 95% CI [26.0%; 34.8%]). The prevalence of insomnia symptoms (dissatisfaction with sleep quantity or quality in the absence of an insomnia disorder diagnosis) observed in the total sample (across diagnostic groups) was 38.4% (164 of 427 patients, 95% CI [33.8%; 43.0%]). Together, 68.8% of the sample (95% CI [66.4%; 73.2%]) were regularly affected by insomnia complaints.

Regarding age, the prevalence of insomnia disorder was 28.6% in the younger half of the patients (61 out of 213 patients, 95% CI [22.5%; 34.7%]) and 32.4% in the older half of the patients (69 out of 213 patients, 95% CI [26.1%; 38.7%]), with patients being divided by the median age of 39.5 years. The prevalence of insomnia disorder was not significantly different between the two age groups (Fisher’s exact test: p = 0.5).

Regarding gender, in the total sample of 427 patients, 225 patients identified as female, 200 as male and 2 as non-binary. The prevalence of insomnia disorder was 36.4% in female patients (82 out of 225, 95% CI [30.1%; 42.7%]) and 24.0% in male patients (48 out of 200, 95% CI [18.1%; 29.9%]). The two patients who identified as non-binary did not meet diagnostic criteria for insomnia disorder. The prevalence of insomnia disorder was significantly higher in female than male patients (Fisher’s exact test: p = 0.006, OR = 1.81, 95% CI [1.19; 2.77]).

The prevalence of insomnia disorder differed between the six participating study centres and ranged from 10.0% to 46.9%. The prevalence of an insomnia disorder diagnosis was 10.0% in Leipzig (95% CI [4.1%; 15.9%]), 27.8% in Mannheim (95% CI [18.0%; 37.6%]), 30.4% in Meiringen (95% CI [19.5%; 41.2%]), 32.5% in Freiburg (95% CI [18.0%; 47.0%]), 46.7% in Bern (95% CI [37.2%; 56.2%]) and 46.9% in Kiel (95% CI [29.6%; 64.2%]).

### Characterization of the study sample

Patients in the total sample were 40.8 ± 15.1 years old. The mean duration of psychiatric illness was 8.6 ± 10.6 years. The majority of the total sample received psychopharmacotherapy (374 patients, 87.6%). Detailed demographic, clinical and psychometric data for the total sample and diagnostic subgroups are depicted in Table 2.

**Table 2:** Descriptive statistics: mean ± SD for continuous variables, and absolute frequency for categorical variables, grouped for psychiatric main diagnosis

Variable	n	F1	n	F2	n	F3	n	F4	n	F6	n	Other	n	Total	P
Demographics															
Age (years)	62	40.7 ± 13.5	54	43.4 ± 14.5	226	42.4 ± 15.1	42	37.5 ± 16.5	29	30.1 ± 11.4	13	37.5 ± 16.1	426	40.8 ± 15.1	0.0005
Gender (%) Female Male Non-binary	21 41 0	33.87 66.13 0.00	25 29 0	46.30 53.70 0.00	124 102 1	54.63 44.93 0.44	25 17 0	59.52 40.48 0.00	25 5 0	82.76 17.24 0.00	6 6 1	46.15 46.15 7.69	225 200 2	52.69 46.84 0.47	0.0004^a^
Education level (%) ≤ *10 years* > *10 years*	17 45	27.42 72.58	18 35	33.96 66.04	30 197	13.22 86.78	13 29	30.05 69.05	16 13	55.17 44.83	6 7	46.15 53.85	100 326	23.47 76.53	< 0.0001
Clinical															
Duration of illness (years after diagnosis)	51	10.1 ± 10.2	29	9.6 ± 11–7	196	9.2 ± 11.0	37	5.4 ± 9.4	24	6.3 ± 8.2	12	5.8 ± 8.5	349	8.6 ± 10.6	0.1675
Age at first main diagnosis (years)	51	30.9 ± 11.5	29	31.5 ± 13.5	197	33.1 ± 14.0	37	31.3 ± 15.0	24	23.9 ± 8.2	12	32.8 ± 18.9	350	31.8 ± 13.7	0.0743
Psychiatric secondary diagnosis (%) No Yes	14 48	22.58 77.42	29 25	53.70 46.30	106 121	46.70 53.30	17 25	40.48 59.52	7 22	24.14 75.86	2 11	15.38 84.62	175 252	40.98 59.02	0.0003
Somatic secondary diagnosis (%) No Yes	31 29	51.67 48.22	31 22	58.49 41.51	99 127	43.81 56.19	24 18	57.14 42.86	18 11	62.07 37.93	7 6	53.85 46.15	210 213	49.65 50.35	0.1734
Sleep medical diagnosis (%) No Yes	60 2	96.77 3.23	46 6	88.46 11.54	187 40	82.38 17.62	36 6	85.71 14.29	21 8	72.41 27.59	11 2	84.62 15.38	361 64	84.94 15.06	0.2198
Current psychopharmacotherapy (%) No Yes	11 51	17.74 82.26	4 50	7.41 92.59	25 202	11.01 88.99	9 33	21.43 78.57	2 27	6.90 93.10	2 11	15.38 84.62	53 374	12.41 87.59	0.2198
Questionnaires															
ISI	59	14.7 ± 6.0	51	13.5 ± 6.9	218	15.7 ± 6.4	40	14.9 ± 7.4	24	16.8 ± 6.0	13	13.8 ± 7.9	405	15.2 ± 6.6	0.2212
PSQI	59	10.8 ± 4.1	48	9.8 ± 4.3	201	11.3 ± 4.3	38	11.3 ± 4.8	25	12.3 ± 3.8	12	10.0 ± 4.4	383	11.1 ± 4.3	0.1611
PHQ-9	61	13.9 ± 6.3	50	13.2 ± 6.5	218	17.1 ± 5.6	40	15.1 ± 6.6	26	16.2 ± 6.1	13	13.7 ± 9.1	408	15.8 ± 6.3	< 0.0001
ESS	61	7.0 ± 4.4	52	7.2 ± 4.6	220	8.0 ± 4.8	40	7.0 ± 4.7	26	8.0 ± 4.3	13	8.7 ± 2.8	412	7.7 ± 4.6	0.4623
FSS	59	4.5 ± 1.5	51	4.3 ± 1.8	217	4.7 ± 1.5	40	4.2 ± 1.8	26	4.4 ± 1.5	13	4.3 ± 1.9	406	4.6 ± 1.6	0.1849
Others															
BMI (kgm^−2^)	60	25.3 ± 4.7	54	27.7 ± 6.0	227	26.1 ± 5.6	42	25.6 ± 6.7	29	27.7 ± 8.0	13	24.5 ± 6.4	425	26.2 ± 5.9	0.1349
# of cigarettes/day	53	18.1 ± 9.3	25	23.3 ± 17.6	106	13.4 ± 9.7	22	14.0 ± 9.3	17	16.5 ± 8.6	4	8.3 ± 3.5	227	15.8 ± 11.0	0.0005

^a^p refers to the test not including the gender category non-binary

F1 substance use disorders, F2 psychotic disorders, F3 mood disorders, F4 neurotic and psychosomatic disorders, F6 personality disorders, and others (including organic psychiatric disorders, eating disorders, and developmental disorders)

*ISI* Insomnia Severity Index, *PSQI* Pittsburgh Sleep Quality Index, *PHQ-9* Patient Health Questionnaire 9, *ESS* Epworth Sleepiness Scale, *FSS* Fatigue Severity Scale

### Frequency and course of insomnia in those with an insomnia disorder diagnosis

In those with a diagnosis of insomnia disorder, the frequency of insomnia symptoms was daily in the majority of patients (62.0%, 95%CI [53.6%; 70.4%]), five to six times per week in 15.5% (95%CI [9.3%; 21.7%]), and three to four times a week in 22.5% (95%CI [15.3%; 29.7%]). Details on the frequency and course of insomnia are displayed in Table 3.

**Table 3:** Frequency and course of insomnia in those with an insomnia disorder diagnosis (N = 130)

Variable	Frequency (%)	95% Confidence interval (%)
Frequency of insomnia symptoms		
Daily	62.0	53.6; 70.4
Five to six times per week	15.5	9.3; 21.7
Three to four times per week	22.5	15.3; 29.7
Insomnia Diagnosis before inpatient stay?		
Yes	11.8	6.2; 17.4
No	88.2	82.6; 93.8
Known insomnia diagnosis in relatives?		
Yes	79.8	76.2; 83.4
No	20.2	16.6; 23.8
Interval from first insomnia symptoms to insomnia diagnosis		
	Mean ± SD (months)	Range
For N = 14 with diagnosis before study	72.0 ± 76.8	1 month to 23 years
For N = 108 with diagnosis in study	135.1 ± 133.6	3 months to 45 years

## Discussion

This study is, to our knowledge, the first interview-based evaluation of the prevalence of insomnia disorder and insomnia symptoms in inpatient psychiatric care. Our main finding is an overall prevalence of insomnia disorder of 30.4% (diagnosis level according to DSM-5) and an additional prevalence of insomnia symptoms of 38.4% (without diagnosis level). Together, this equals a prevalence of sleep dissatisfaction of 68.8%. These findings underline the importance of the problem and the need for systematic diagnosis in inpatient psychiatry, as a prerequisite for optimized treatment and health care. A particular strength of our study is the use of comprehensive structured diagnostic interviews by experienced clinicians.

A previous questionnaire-based study in 203 inpatients in psychiatric care using the Insomnia Severity Index as a screening for insomnia disorder found a prevalence of 67.4% [19]. This prevalence equals the interview-based results from our study for insomnia disorder and insomnia symptoms together, underlining that questionnaire-based screenings often lead to inflated prevalence rates and are not suitable for a distinction between a disorder and symptom level [13]. Based on diagnostic interviews, in the adult general population, approximately 10% meet the criteria for an insomnia diagnosis, and another 20% experience occasional symptoms of insomnia not meeting the full diagnostic criteria [13]. In outpatients with a psychiatric diagnosis, the prevalence was estimated at 31.8% in an interview-based study [12]. Together with our findings, the current literature demonstrates that approximately one third of those diagnosed with a psychiatric disorder fulfill the criteria for comorbid insomnia disorder—independent of whether they are treated in an outpatient or inpatient setting. This prevalence is more than three times higher than in the general population. Together with the detrimental impact of insomnia on quality of life and mental health, this underlines the importance of insomnia diagnosis and treatment in psychiatry.

Overall, our pattern of results suggests that insomnia disorder is a highly prevalent problem in inpatient psychiatric care—across diagnostic entities, age groups, genders, and relatively independent of other demographic variables. More specifically, the prevalence of insomnia disorder is high in inpatients with substance use disorders, psychotic disorders, mood disorders, anxiety disorders and personality disorders. For the other diagnostic categories including organic psychiatric disorders, eating disorders and attention deficit disorders, the number of patients in our study was too low to draw solid conclusions. While the prevalence of insomnia disorder was higher in women than in men (36.4% vs. 24.0%), the prevalence was overall high in both genders. We did not find statistically significant differences in the prevalence between the younger and older half of patients. These observations suggest that the high prevalence of insomnia disorder is not limited to specific diagnostic or demographic groups, but rather that insomnia disorder represents a common comorbidity among patients receiving inpatient psychiatric care. Despite the high prevalence, insomnia disorder was pre-diagnosed in only 11.8% of those who received an insomnia disorder diagnosis in our study. The remaining 88.2% received this diagnosis for the first time in our study. This suggests that insomnia disorder is under-diagnosed, which likely results in under-treatment or treatment outside of guideline recommendations. However, since the data regarding a previous diagnosis were based on patient reports, we cannot exclude that these results are biased—patients may have forgotten about the term “insomnia disorder” or may actually not have fulfilled diagnostic criteria at a previous point in time.

The distribution of diagnostic groups in our sample reflects the current situation in German and Swiss inpatient psychiatric care, with affective disorders being the most common reason for hospital admission, a relatively high number of patients with substance use disorders, psychotic disorders and anxiety/obsessive compulsive disorder, and a small number of patients with organic psychiatric disorders, eating disorders, attention deficit hyperactivity disorders (ADHD) and developmental disorders (as the primary diagnosis). A potential reason for the low prevalence of organic psychiatric disorders and developmental disorders may be that patients under custody were not included in our study. In our study, patients were grouped according to their primary diagnosis, which we defined here as the primary disorder being responsible for the admission to psychiatric inpatient treatment. This way of categorizing patients does not reflect comorbidity—many patients had comorbid diagnoses but are reflected only with their primary diagnosis in the current analysis. The low percentage of patients with ADHD and eating disorders may reflect that these are frequently comorbid with other psychiatric disorders including depression but are rarely the main cause for a psychiatric inpatient stay. Accordingly, our study does not allow for a prevalence estimate of insomnia disorder in under-represented but important diagnostic categories such as ADHD and eating disorders. This gap in knowledge needs to be addressed in future research.

Several limitations of the present study need to be discussed. First, our findings cannot be generalized to patients admitted involuntarily or under custody, because they were excluded from our sample. As it seems likely that this patient group has more severe psychiatric conditions, this might have biased our results in the direction that the prevalence of insomnia disorder and insomnia symptoms might be higher than reported. Second, the number of randomly selected participants per week was determined by clinician workload, instead of a fixed number or proportion. This might have compromized the randomization process such that severely ill patients who contribute to high workload might be underrepresented. This, in turn, might have led to an underestimation of the prevalence of insomnia in our study. However, while we acknowledge that workload and prevalence might in part be correlated, we expect this effect to be rather moderate because patients were recruited throughout large hospitals, and their characteristics are probably in part independent of the work load of an individual clinician. Third, while basic standards were ensured across study sites, including a written interview guide and meetings to discuss diagnostic procedures, full standardization was not achieved in this pragmatic clinical project and smaller variations between study centers regarding the procedure of the interview and interpretation of diagnostic criteria might have occurred. This potentially contributed to the relatively high variance in prevalence of insomnia diagnosis and insomnia symptoms between the participating study centers.

In our study, the “insomnia symptoms” group is a heterogeneous group including both those who did not fulfill criteria for insomnia disorder in terms of severity or duration, and those whose insomnia symptoms were explained through another disorder (or both). In the process of diagnosing insomnia in those with a psychiatric diagnosis, clinicians need to differentiate between insomnia disorder as a comorbidity, and insomnia as a symptom of the psychiatric disorder. This is especially relevant in disorders for which sleep difficulties are listed as a diagnostic symptom—such as affective disorders and post-traumatic stress disorder. The distinction between disorder and symptoms is not given by nature but relies on the clinical evaluation and definition. To guide decision making and to unify the diagnostic approach, we added three questions beyond the DSM-5 criteria. These questions reflect that a comorbid insomnia diagnosis, in contrast to insomnia symptoms in the context of another disorder, appears especially justified when the time course of the two disorders is at least partly independent (time criterion, e.g. with insomnia present outside of an episode of another disorder), when the severity of the insomnia is so high that it appears not sufficiently explained by the severity of the other disorder (severity criterion), and when a patient’s cognitive and emotional focus on sleep and insomnia is high (cognitive criterion). Despite our efforts to guide decision making, the prevalence of insomnia disorder diagnosis varied between participating centers. This may, be due to different patient characteristics or differences in the diagnostic procedures.

The proposed diagnostic principle reflects—in the context of fluid boundaries—a pragmatic approach (as for other mental disorders). The high rate of comorbidity between insomnia disorder and psychiatric disorders suggests that there may be a shared pathophysiology. The pathophysiology, however, remains to be further determined and likely includes both biological, including genetic factors [20], and psychological factors. In the absence of a mechanistic understanding, the proposed approach serves to identify those who will likely benefit from a specific treatment of insomnia, in addition to the treatment of the comorbid psychiatric disorder. Particularly, the high prevalence of insomnia disorder and insomnia symptoms in inpatient psychiatric care reflects a call for action in terms of diagnosis and treatment. While a majority of patients in psychiatry report sleep problems, a systematic diagnosis and differential diagnosis for different sleep disorders is rather the exception than the norm. In clinical reality, insomnia treatment frequently consists of hypnotic medication. While benzodiazepines and benzodiazepine receptor agonists may play a role in the treatment of acute and severe insomnia in psychiatry, their longer-term use bears the risk of the development of tolerance and dependency [21]. Other medications frequently prescribed for insomnia include sedating antidepressants, antipsychotics and newer orexine receptor antagonists. While their risk for tolerance and dependency is lower than for benzodiazepines, they still bear a risk of side effects including weight gain and sedation for antidepressants and antipsychotics, and headaches and fatigue for orexin receptor antagonists.

Importantly, evidence-based treatment guidelines for insomnia disorder endorse CBT-I as the first-line treatment for patients with insomnia disorder—including those with a comorbid psychiatric diagnosis [7, 8]. CBT-I is a treatment package including psychoeducation, restriction of time in bed, stimulus control, relaxation, and cognitive restructuring. One of the major mechanisms of action is an increase of homeostatic sleep pressure through a reduced time spent in bed, thereby improving sleep quality. Despite this recommendation, CBT-I or adaptations are insufficiently implemented in inpatient psychiatric care [22]. Recent work suggests that adaptations of CBT-I for patients with comorbidity can be successfully implemented in inpatient psychiatric care [22–24]. Different adaptations of CBT-I share a transdiagnostic approach and a focus on behavioral elements of CBT-I, most prominently the restriction of time in bed [23, 24]. For instance, *SLEEPexpert* is a behavioral treatment program for patients with insomnia disorder in complex medical settings, including inpatient psychiatric care [23]. For this purpose, elements of CBT-I have been adapted to the needs of patients and health care teams, with the idea that the program can be implemented using existing resources. The main therapeutic intervention of SLEEPexpert is the restriction of time in bed and the implementation of a new, individualised “sleep window”—that is, bedtimes adapted to the current individual sleep ability of the patient. Preliminary data demonstrate that the program is feasible and potentially effective in an acute psychiatric setting [23].

In summary, the results of the present study demonstrate a high prevalence of around 30% of insomnia on a diagnosis level and an additional 38% prevalence of insomnia symptoms not fulfilling diagnostic criteria in inpatient psychiatric care. Future goals include replication and extension of the current work, with a focus on gathering reliable and valid, interview-based diagnosis estimates for insomnia disorder across medical care settings. Further crucial steps are the implementation of a systematic diagnostic process for insomnia disorder and treatment in various settings including inpatient psychiatric care. Treatment should, in line with current guidelines, particularly center on CBT-I or adaptations and contribute to reducing over-treatment with hypnotic medication.

## Data Availability

Data are available upon reasonable request to the corresponding author.
